# HDAC-driven mechanisms in anticancer resistance: epigenetics and beyond

**DOI:** 10.20517/cdr.2024.103

**Published:** 2024-11-20

**Authors:** Martina Minisini, Martina Mascaro, Claudio Brancolini

**Affiliations:** Laboratory of Epigenomics, Department of Medicine, Università degli Studi di Udine, Udine 33100, Italy.

**Keywords:** HDACs, chemotherapy, hormone deprivation, acetylation, palbociclib, PARP, DNA damage

## Abstract

The emergence of drug resistance leading to cancer recurrence is one of the challenges in the treatment of cancer patients. Several mechanisms can lead to drug resistance, including epigenetic changes. Histone deacetylases (HDACs) play a key role in chromatin regulation through epigenetic mechanisms and are also involved in drug resistance. The control of histone acetylation and the accessibility of regulatory DNA sequences such as promoters, enhancers, and super-enhancers are known mechanisms by which HDACs influence gene expression. Other targets of HDACs that are not histones can also contribute to resistance. This review describes the contribution of HDACs to the mechanisms that, in some cases, may determine resistance to chemotherapy or other cancer treatments.

## INTRODUCTION

Much progress has been made in the detection and treatment of cancer. Unfortunately, there are still several key problems to be solved, including very aggressive specific cancers, metastasis, and resistance to therapy^[1,2]^. Cancer therapy involves multiple strategies and approaches, from surgery to immunotherapy. Chemotherapy is still part of several protocols in cancer treatment or adjuvant therapies^[3]^. Several chemotherapeutic agents work by damaging essential cell functions or triggering uncontrollable cell stress, leading to cell death^[4]^. All these agents have certain limitations due to their toxicity to non-tumor cells and the emergence of resistance^[5]^. Drug resistance is one of the main causes of failure of cancer therapies and can also lead to multidrug resistance (MDR)^[2]^. The first findings on resistance go back to Luria and Delbruck, who described that genetic mutations in bacteria make them resistant to viral infection^[6]^. Goldie and Coldman later transferred this concept to the resistance of cancer to therapeutic drugs, which is due to random mutations in cells^[7]^.

Depending on the mechanism, chemoresistance can be intrinsic, if it occurs before treatment due to genetic and epigenetic factors, or acquired, if it arises during treatment in previously susceptible tumor cells^[8]^. Various studies have described different molecular mechanisms that play a role in drug resistance: transport pumps, DNA damage and repair, oncogenes and tumor suppressor genes, epithelial-mesenchymal transition (EMT), *etc.* In this review, histone deacetylases (HDACs) are discussed with a focus on class I and IIa and their involvement in the development of mechanisms of drug resistance. Regulation of lysine acetylation turnover by HDACs can affect various cellular responses, including drug resistance phenotypes. Resistance can be influenced by changes in gene expression, which is determined by the control of chromatin accessibility (epigenetics) or, for example, by the activity of transcription factors (TFs) that are subject to acetylation. In addition, other biological functions not related to gene expression may also be under the control of lysine acetylation turnover. Importantly, a repertoire of different HDAC inhibitors (HDACi) is available that may counteract HDAC-induced drug resistance.

## THE HDAC WORLD IN BRIEF

Acetylation is an ancient and very simple post-translational modification (PTM) of amino acids, but its effects on cell fate are impressive. Two enzyme families act antagonistically in the control of lysine acetylation: the lysine acetyltransferases (KATs) and the lysine deacetylases (KDACs). As histones are important and well-studied substrates for these enzymes, they are also referred to as histone acetyltransferases (HATs) and HDACs. In this chapter, we would like to give a brief introduction to the world of HDACs and highlight some important features.

HDACs represent a heterogeneous family of 18 enzymes that can be divided into five different classes/subclasses^[9,10]^. They are responsible for removing the acetyl group but also other small hydrophobic acyl groups, such as formyl, propionyl, butyryl, malonyl, succinyl, glutaryl, crotonyl, and 2-hydroxyisobutyryl from ε-amino group of lysines^[9,11,12]^. The first distinction, based on the catalytic mechanism, the required cofactor, and the structure of the catalytic pocket, is between zinc- or metal-dependent and NAD^+^-dependent enzymes^[13]^.

Eleven are the metal-dependent HDACs, which are divided into three classes based on sequence and structural homologies. Class I includes HDAC1, 2, 3, and 8, which are largely nuclear enzymes. HDAC1 and HDAC2 are much closer to each other and often coassemble in different repressive complexes^[14]^. Like HDAC1 and 2, HDAC3 forms multiprotein complexes that are important for the maturation of full catalytic activity. HDAC8 is the more distant member and does not need to assemble into protein complexes to develop full catalytic activity. HDAC1, 2, or 3 often has a strong influence on cell proliferation^[15,16]^.

**Figure 1:**
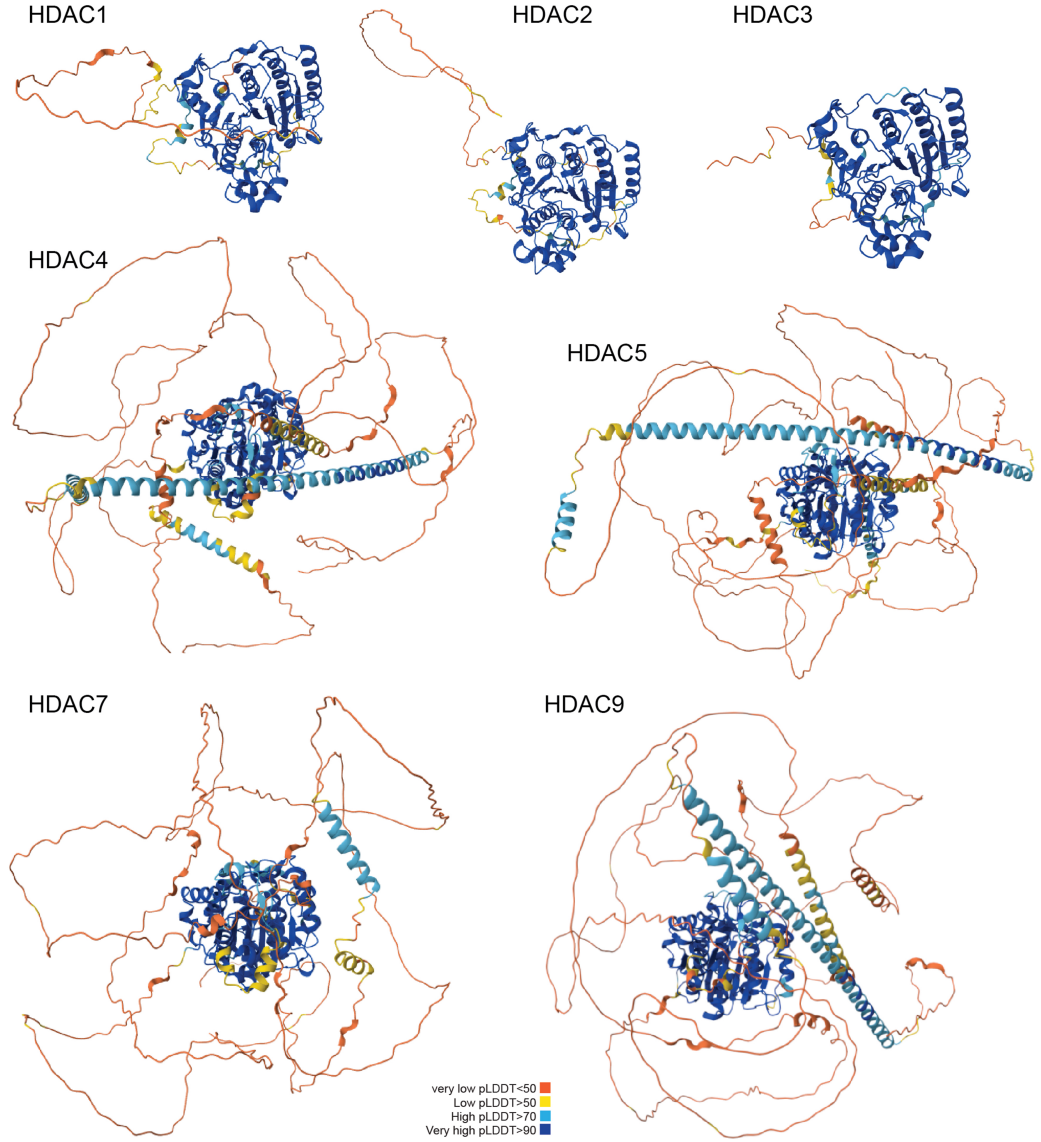
AlphaFold prediction of Class I and Class IIa structures discussed in this review (Q13547; Q92769; O15379; P56524; Q9UQL6. Q8WUI4; Q9UKV0). The different views were similarly oriented, taking as reference the eight-stranded parallel β-sheet of the common deacetylase domain. The colors indicate the different per-residue confidence scores (pLDDT). Some regions below 50 pLDDT may be unstructured in isolation. For details, see https://alphafold.ebi.ac.uk. pLDDT: Predicted Local Distance Difference Test.

HDAC4, HDAC5, HDAC7, and HDAC9 form class IIa, which is distinct from class IIb. Common features of class IIa are the intense regulation of nuclear-cytoplasmic shuttling by phosphorylation and the acquisition of the Tyr/His mutation in the catalytic pocket in vertebrates. This amino acid substitution minimizes/abrogates KDAC activity. In addition, class IIa HDACs show the presence of an extensive amino-terminal region dedicated to protein interaction, including the binding of co-repressors and TFs [Figure 1], of which the members of the myocyte enhancer factor 2 (MEF2) family are best characterized^[17]^. In vertebrates, class IIa shows very low deacetylase activity toward acetyl-lysines, but their ability to bind with repressive class I complexes allows deacetylase activity in trans^[18,19]^. For this reason, it has been proposed that class IIa acts as an acetyl-lysine reader^[20-22]^. Class IIa, apart from HDAC7, also possesses a glutamine-rich domain that enables homo-/heterodimerization [Figure 1] and still unexplored influences on chromatin structure^[23]^.

Class IIb essentially comprises the cytosolic enzymes HDAC6 and HDAC10. HDAC6 has several target proteins that are not histones, such as α-tubulin and Hsp90, and can regulate their stability by deacetylation^[24,25]^. In contrast, HDAC10 is a polyamine deacetylase with a very high substrate specificity. The structure of the active site is specific for the hydrolysis of N8-acetylspermidine and disfavors the hydrolysis of acetyl lysine^[26,27]^.

The NAD^+^-dependent HDACs are known as sirtuins. They form class III and are not discussed in this overview. Finally, HDAC11 alone constitutes the class IV. HDAC11 is a potent lysine defatty-acylase rather than a deacetylase and makes important contributions to the regulation of the immune system.

For a discussion of the general and specific aspects of the various HDACs, we refer the reader to previously published reviews^[9,10,15,22,28-31]^.

In summary, zinc-dependent HDACs 1, 2, 3, which are part of various multiprotein complexes and, in some cases, interact with class IIa HDACs, serve as master regulators of histone acetylation and strong candidates for modulating epigenetic resistance to anticancer therapies. Given that these HDACs are components of different multiprotein complexes whose composition - both in terms of stoichiometry and individual components - varies depending on the neoplastic context, HDACs can either promote drug resistance or act antagonistically. Each of these aspects requires detailed investigation.

## RESISTANCE TO CHEMOTHERAPY: CISPLATIN

Cisplatin is a metal-based chemotherapeutic agent used to treat various types of cancer^[32,33]^. Cisplatin binds to DNA and leads to the formation of DNA-platinum adducts, which initiate the DNA damage response (DDR)^[33,34]^. The efficacy of this approach decreases over time due to the emergence of drug resistance^[32,33]^. Resistance to cisplatin can arise through many different mechanisms^[35]^. Recently, class I HDACs have also been found to be responsible for cisplatin resistance/sensitization. In MCF7 breast cancer cells, TRIM46-dependent degradation of HDAC1 enhances the expression of genes involved in both DNA replication and DNA repair^[36]^. Proteasomal-dependent degradation of HDAC1 increases the expression of genes involved in DNA repair, such as breast cancer 1 gene (*BRCA1*)^[36]^, which is involved in chemoresistance to various drugs^[36-38]^. The chemosensitivity of cisplatin is also regulated in laryngeal cancer cells by HDAC1 via the modulation of interleukin-8 expression^[39]^. Differently, in non-small cell lung cancer (NSCLC), an epigenetic mechanism under HDAC1 control regulates cisplatin resistance by suppressing the expression of ornithine decarboxylase antizyme 1 (OAZ1)^[40]^. OAZ1 triggers the degradation of ornithine decarboxylase and suppresses the synthesis of polyamines^[41]^, but how this leads to resistance remains to be clarified. The role of HDAC2 in chromatin remodeling at the site of DNA lesions, which could contribute to the regulation of chemoresistance, is interesting but remains poorly understood^[18,42,43]^.

Surprisingly, the role of HDAC3 in cisplatin resistance is not well understood^[44]^, but its contribution when complexed with class IIa should be considered, and indeed, some reports have suggested a role for HDAC4 in regulating cisplatin resistance. Stronach *et al.* have described that ovarian cancer cells resistant to cisplatin have upregulated expression of both HDAC4 and STAT1 and that downregulation of HDAC4 leads to platinum sensitization, increased caspase activation and apoptosis. In addition, HDAC4 is overexpressed in clinical samples that exhibit platinum resistance. HDAC4 and STAT1 can interact through co-immunoprecipitation, and HDAC4 deacetylates STAT1, causing its phosphorylation at Y701. This regulation triggers the nuclear translocation of STAT1 and the activation of its transcriptional program, leading to platinum resistance^[45]^. Further confirmation using additional methods such as mass spectrometry should help to validate this interaction further. Since HDAC3 is the predominant HDAC4 partner conferring enzymatic activity to the complex, its silencing in the regulation of STAT1- and HDAC4-dependent deacetylation should be investigated.

In gastric cancer cells, analysis of the cisplatin pangenome response identified HDAC4 as the major repressed epigenetic regulator. HDAC4 inhibits the cytotoxicity of cisplatin and favors chemoresistance. Its expression is high in gastric tumors compared to healthy tissue, while HDAC4 mutations correlate with a good prognosis^[46]^. This effect is thought to be partially dependent on TP53^[46]^. Furthermore, the involvement of HDAC4 in the regulation of H2K120ac status at the site of DNA lesions, as well as its influence on the efficiency of homology-directed repair via HDAC2, may also contribute to this resistance^[18]^.

Mutations in *TP53* that promote cell apoptosis, senescence, and cell cycle arrest and are associated with platinum chemoresistance are common in ovarian cancer^[47,48]^. Reintroduction of *TP53* into the ovarian adenocarcinoma cell line SKOV-3, which is characterized by its deletion, leads to upregulation of pro-apoptotic proteins and reduces cis-platinum resistance^[49]^. Importantly, in this context, *TP53* induction decreased *HDAC4* expression while also increasing HDAC4 phosphorylation and its accumulation in the cytoplasm - both of which diminish its repressive influence. As a result, an increase in total histone 3 acetylation was observed^[49]^. Although the relationships between HDAC4 and HIF-1a have not been fully explored, Zhang *et al.* reported that they form a complex that regulates autophagy and apoptosis controlled by TP53^[49,50]^. In ovarian and lung cancer cells, this complex is crucial for cis-platinum resistance. Mechanistically, overexpression of *HIF-1a* inhibits *ATG12* and *BAX*, but enhances *BCL2* expression. Autophagy is directly mediated by HDAC4 through deacetylation of the TF CREBZF, which is responsible for the transcription of *ATG2* and possibly *ATG12*. Under normoxic conditions, *TP53* leads to a massive decrease in HIF-1a, while expression of HDAC4 and HIF-1a leads to cis-platinum resistance and worsened overall survival in ovarian cancer patients^[49]^.

**Figure 2:**
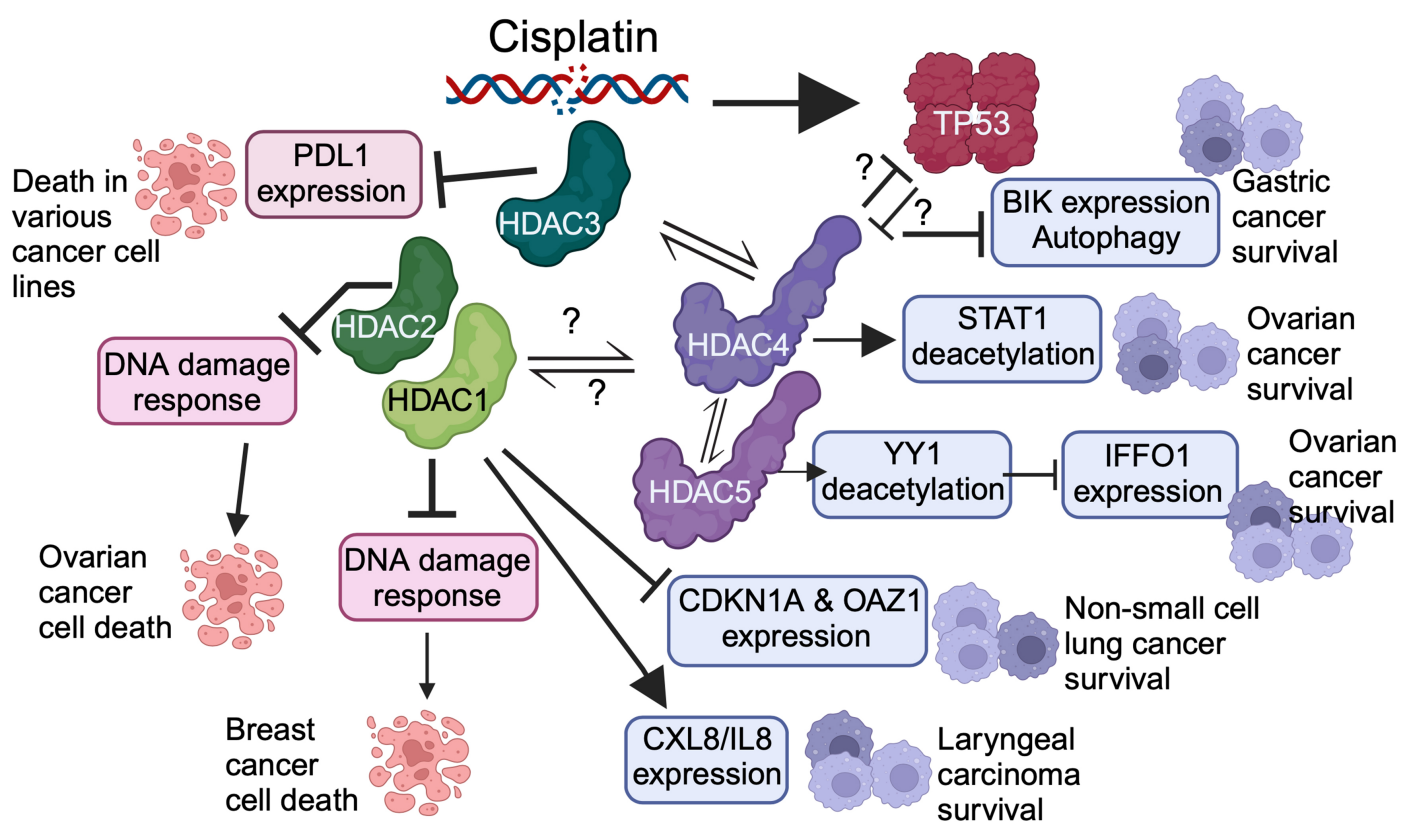
Summary of reported roles and mechanisms of class I and class IIa HDACs in different cancer cell lines that respond to treatment with cisplatin. Mechanisms of resistance are shown in purple, while mechanisms of susceptibility are shown in light orange. The relationships between HDAC4 and TP53 are poorly defined (question marks). In addition, the existence of different multiprotein complexes with class I and IIa HDACs is also considered. BIK is a BH3-only pro-apoptotic BCL2 family member. For details, please refer to the text. HDACs: Histone deacetylases; BIK: BCL2 interacting killer; BCL2: B-cell CLL/lymphoma 2.

Another example of the involvement of class IIa HDACs in cisplatin resistance in ovarian cancer is the tumor suppressive role of intermediate filament family orphan 1 (IFFO1). IFFO1 plays an important role in the regulation of tumor progression and immune infiltration^[51,52]^. It is poorly expressed in ovarian cancer tissue, as well as other cancer tissues, and cisplatin-resistant cells are characterized by a low *IFFO1* level. The regulatory mechanism of IFFO1 depends on the interaction between HDAC5 and the TF YY1. HDAC5 binds to the *IFFO1* promoter via YY1, leading to its deacetylation and a reduction in transcription^[53]^. Since the interaction between YY1 and HDAC5 was observed also in other studies^[54-56]^ and HDAC5 expression is altered in ovarian cancer, this result is of particular relevance^[57]^. Figure 2 summarizes the effects and mechanisms of class I and IIa HDACs that may influence the response of cancer cells to treatment with cisplatin.

## OTHER GENOTOXIC DRUGS

Resistance to other genotoxic drugs has also been reported to depend on class I and IIa HDACs. In multiple myeloma (MM), inhibition of HDAC1 leads to a re-sensitization of cells to the proteasome inhibitor bortezomib (BTZ) and to a reduction in DNA repair potential, thus potentially increasing the susceptibility to genotoxic stress. The heterochromatin protein 1 (HP1) family α, β, and γ are readers of H3K9me2/3^[58]^. HP1α and β are distributed along heterochromatic regions (centrosomes and telomeres), while HP1γ is associated with both euchromatin and heterochromatin, in regions with actively transcribed genes that play a role in transcriptional elongation^[59]^. HP1γ acts as a platform for histone modifiers in association with HDAC1^[60]^. In MM, HDAC1 interacts directly with the protein HP1γ and deacetylates it at Lys 5 to improve the stability of the protein and its nuclear condensation. This improves DNA repair by forming a complex with MDC1 and promotes chromosomal accessibility of genes that determine MM cell survival and BTZ resistance^[61]^. A mechanism that should also be evaluated for the response to other genotoxic treatments.

Doxorubicin is an anthracycline antibiotic that acts by inhibiting topoisomerase IIa (TOPO2A), thereby causing an accumulation of double-strand breaks (DSBs). It was first isolated from *Streptomyces peucetius* by Arcamone in 1969^[62]^. Resistance to doxorubicin is mainly related to the overexpression of ABC efflux pumps. Other mechanisms include increased expression of *TOPO2A* or anti-apoptotic BCL2 proteins^[63]^. Reduced oxygen species (ROS) and a change in redox activity/status may also promote resistance to doxorubicin as well as other chemotherapeutic agents^[64]^.

A non-epigenetic role of HDAC3 in acute myeloid leukemia (AML) responsible for resistance to anthracyclines and cytarabine has been reported. Deletion or inhibition of *HDAC3* impairs DNA repair, which occurs mainly via the AKT pathway^[65]^. This pathway is involved in cell survival and proliferation in leukemia, where it is often activated and associated with a poor prognosis^[66]^. Increased AKT signaling is associated with the onset of chemoresistance^[67]^. Treatment with Ara-C in combination with doxorubicin leads to increased expression of HDAC3, which interacts with AKT and deacetylates it at the level of Lys 20. This modification leads to its phosphorylation and increased activity^[65]^. Through this mechanism, AML cells activate the resolution of DNA damage. Finally, treatment with the HDAC3 inhibitor (RGFP966) reverses Ara-C and doxorubicin resistance^[65]^.

In lung cancer-derived cells with exogenous overexpression of cancer-regulated gene 2 (*CUG2*), there is upregulation of EGFR and consequent resistance to doxorubicin treatment due to increased expression of antioxidant proteins such as MnSOD, Foxo1, and Foxo4 and the MDR genes *MRP2* and *BCRP*. EGFR causes the activation of STAT1, a known factor of doxorubicin resistance, which was downregulated by the non-selective HDACs inhibitor TSA thanks to increased acetylation^[68]^. *HDAC4* is highly expressed in CUG2 cells, and downregulation of *HDAC4* leads to doxorubicin sensitization through a decrease in antioxidant proteins and increased apoptosis^[69]^.

## ANTIMETABOLITES

Antimetabolites interfere with tumor growth by inhibiting pyrimidine and purine biosynthesis, which leads to a blockade of DNA synthesis and triggers DNA strand breaks and apoptosis in highly proliferating cancer cells^[70]^. Antimetabolites are divided into purine analogs (6-mercaptopurine), pyrimidine analogs [5-fluorouracil (5-FU), gemcitabine, azacitidine], and antifolates (methotrexate). Azacitidine and its derivatives are epigenetic drugs that inhibit DNA methyltransferases (DNMT)^[71]^. In addition to the classical resistance mechanisms, antimetabolites can cause an overexpression of thymidylate synthase, an accumulation of deoxyuridine monophosphate, and a downregulation of 5,10-methylenetetrahydrofolate^[72]^. Gemcitabine resistance is associated with changes in proteins involved in its metabolism^[73]^. Methotrexate resistance is caused by changes in dihydrofolate reductase and retention of the drug in the intracellular space^[74]^.

5-FU is the main therapy for colorectal cancer (CRC). Unfortunately, patients often became resistant to this treatment^[75,76]^. Dihydropyrimidine dehydrogenase (DPD) can rapidly degrade 5-FU and confer resistance^[77]^. The sphingosine kinase SphK2 produces sphingosine-1-phosphate (S1P) in the cell nucleus, which has been shown to inhibit the activities of Hdac1 and 2^[78]^. High *SPHK2* expression in CRC cell lines promotes an overall increase in H3K56 acetylation as determined by whole genome analysis. The increase in H3K56ac also affects an exon of DPD and correlates with its increased expression. Therefore, HDAC1 and 2 repress the transcription of DPD and the degradation of intracellular 5-FU. In this context, HDAC1 and 2 are considered epigenetic regulators that counteract resistance to 5-FU^[79]^. However, contrary results have also been reported in various contexts. Resistance to 5-FU was observed in the CRC cell line RKO, which is negative for HDAC2. This benefit was associated with a higher efficiency of ATM signaling and possibly enhanced DNA repair activity, but with an undefined mechanism^[80]^. Studies on class IIa and 5-FU resistance date back to 2010, when it was reported that HDAC7 affects HIF-1a-induced resistance to 5-FU-induced apoptosis in lung cancer cells^[81]^. In osteosarcoma, miR-140 has been reported to contribute to chemoresistance to methotrexate and 5-FU. The proposed mechanism involves suppression of HDAC4 expression and consequent cell cycle arrest in G1 and G2 with a reduced proliferation rate^[82]^. Other studies have also indicated a contribution of HDAC4 to 5-FU resistance in CRC^[83]^. In summary, although there is some evidence for a possible contribution of class IIa to 5-FU resistance, the mechanisms involved still need to be clarified and further studies are required.

HDAC4, together with HDAC7, may also contribute to resistance to gemcitabine in pancreatic ductal adenocarcinoma (PDAC). Knockdown (KD) of these two HDACs enhances gemcitabine cytotoxicity and apoptosis both *in vitro* and *in vivo* in mouse models of PDAC. Interestingly, this activity is caused by MARK2-dependent phosphorylation and the cytosolic accumulation of deacetylases. As discussed below, this cytosolic accumulation of class IIa HDACs also influences resistance to taxanes^[84]^.

## TAXANES

Taxanes are a class of chemotherapeutic agents that influence microtubule dynamics. In highly proliferating cells, they primarily cause mitotic arrest and, ultimately, cell death by mitotic catastrophe^[85]^. The taxanes include paclitaxel (PTX), docetaxel (DTX), and cabazitaxel. They are often used to treat metastatic breast cancer, non-small lung cancer, prostate, ovarian and bladder cancer. PTX and DTX bind to a hydrophobic cleft in β-tubulin^[86]^. PTX is the most used drug for the treatment of triple-negative breast cancer (TNBC)^[87,88]^. Patients may develop resistance through changes in cell survival, changes in tubulin, such as mutations that reduce binding to PTX, PTMs, or increased expression of MDR efflux transporters^[89,90]^.

In NSCLC, HDAC1 is critical for resistance to PTX^[91]^. HDAC1 can form a complex with SIN3A, a core component of the histone deacetylation activity-associated transcriptional repressor complex^[92]^, and C3b, the active fragment of complement component 3 (C3)^[93,94]^. This repressive complex controls the downregulation of the antiproliferative gene *GADD45D*^[95]^ by histone deacetylation and thus leads to PTX resistance^[96]^.

The involvement of HDAC1 in the maintenance of resistance to taxanes has been confirmed for DTX and again for NSCLC, but the proposed mechanism is different and involves miR-200b. Of interest is the definition of the epigenetic switches that control *HDAC1* transcription. Here, *HDAC1* is repressed after H3K27me3 modification of its promoter by SUZ12, the catalytic component of polycomb repressive complex 2 (PRC2)^[97-99]^. *HDAC1* suppression leads to an increase in miR-200b levels, which are known to reverse DTX resistance^[100]^. SUZ12 has been reported to be recruited to the HDAC1 promoter by the lncRNA *MARCKSL1-2*^[99]^. Interestingly, a contribution of HDAC3 to taxane resistance has also been observed in some studies^[101,102]^. It will be important to study whether a complex among HDAC3 and class IIa HDACs is required to confer resistance.

Using a single-guide RNA screen to assess genes involved in PTX resistance, class IIa HDAC9 and its truncated amino-terminal isoform MEF-2 interacting transcription repressor (MITR) were identified among the top hits^[103]^. MITR is highly expressed in the PTX-resistant TNBC cells MDA-MB-231, and KD of MITR results in an impressive reduction in the IC50 of PTX compared to control cells or cells with HDAC9 KD. This outcome likely reflects the relative abundance of the two isoforms in this cell line. Overexpression of MITR enriches the JAK/STAT3 pathway and leads to an increase in IL11 production. Interestingly, silencing *IL11* abrogates PTX resistance in MITR-overexpressing cells. Mechanistically, MITR interacts with MEF2A and abolishes its repressive influence on the *IL11* promoter^[104]^. The mechanisms underlying MITR’s influence on *MEF2A* and *IL11* expression are unclear, as is the potential involvement of other HDACs. This hypothesis requires further studies.

Microtubule affinity-regulating kinases (MARK1-4) are important regulators of cell polarity and cell division. They are upregulated in various pathological conditions such as cancer^[105,106]^. High MARK2 expression correlates with poor prognosis in patients with pancreatic cancer and with PTX resistance. In response to PTX treatment, MARK2 phosphorylates class IIa HDACs (mainly HDAC4 and HDAC7 in this study) and promotes their cytoplasmic localization. PTX resistance of MARKs depends on the phosphorylation of HDAC4/7, which in turn stimulates YAP activity by modulating the LATS2/YAP complex. KD of *HDAC4* and *HDAC7* abolishes PTX resistance in PANC-1 cells^[84]^. Additional mechanisms have been proposed for the regulation of DTX resistance by HDAC4 based on the suppression of H3 acetylation of the miR-200b promoter^[100]^.

## HORMONE THERAPY

Hormone therapy uses the natural function of hormones as chemical messengers produced by endocrine organs to treat hormone-dependent cancers such as breast, prostate, and ovarian cancer. It includes hormone analogs, inhibitors of hormone receptors or hormone synthesis^[107]^. The first hormonal agent used in clinical trials was tamoxifen^[108,109]^. Endocrine therapy, especially the estrogen antagonist tamoxifen, has been shown to improve the survival of patients with estrogen receptor-alpha-positive breast cancer. However, the occurrence of tamoxifen resistance is an increasing obstacle to the effectiveness of the therapy. Endocrine resistance is mainly due to aberration of estrogen/progesterone receptor signaling pathways, PTMs, epigenetic alterations of estrogen receptor 1 (ESR1), increased signaling through the tyrosine kinase receptor, and alteration of cell cycle progression^[110]^.

Epigenetic remodeling may be responsible for resistance to endocrine therapies^[111]^, and several studies have shown that HDACi can reverse such resistance^[112-115]^. However, in some clinical trials with different HDACi and exemestane (AI-aromatase inhibitor), contrasting results were obtained, with an improved survival rate not always observed^[116]^. In addition, repressive HDAC complexes can associate with estrogen receptors (ERs) and contribute to their transcriptional output^[117-125]^.

To solve this problem, Zhou *et al.* developed an AI-resistant cell model and found that downregulation of serine protease inhibitor serpin family A member 3 (*SERPINA3*), a target gene of ESR1, induces endocrine resistance^[126]^. Its downregulation increases the expression levels of ankyrin repeat domain containing 11 (*ANKRD11*), which interacts directly with HDAC3. Deacetylation of H3K9ac, which occurs through HDAC3 in complex with ANKRD11, promotes AI resistance. The use of RGFP966, an HDAC3-specific inhibitor, or *HDAC3* silencing abolishes AI resistance^[125]^. However, the epigenomic elements regulated by HDAC3 in this tumor context are still unknown, and we should also consider the contributions of HDAC3 as a partner of ERs^[127,128]^. HDAC2, HDAC5, and HDAC9 have also been associated with tamoxifen resistance^[129,130]^.

Of particular interest is that impairment of HDAC1/2 can cause basal-like cells to adopt a luminal A-cell phenotype with increased ER expression and increased sensitivity to tamoxifen treatment^[131]^.

The TF, stem cell-related SOX9, is responsible for tamoxifen resistance, tumor invasion, and metastasis^[132,133]^. One of the regulators of SOX9 is SIRT1. It causes SOX9 deacetylation, which is required for its increased localization in the nucleus. SOX9 is localized in the nucleus in ER+ breast cancer cells, and its level is increased in cells resistant to tamoxifen^[130]^. Similarly, the deacetylase domain of HDAC5 physically interacts with SOX9 via its HMGB domain and triggers its deacetylation and nuclear localization. This leads to tamoxifen resistance thanks to the SOX9 transcription program^[130]^. In addition, HDAC5 is regulated by C-MYC, which directly controls its expression in breast cancer cells. C-MYC is a known trigger of tamoxifen resistance in breast cancer^[134]^. Consequently, switching off HDAC5 significantly reduces tamoxifen resistance^[129]^. Similarly, HDAC9 has been associated with tamoxifen resistance. Its expression correlates with poor response to 4-hydroxy-tamoxifen (OHTam) and poorer prognosis in patients treated with OHTam. Its ectopic overexpression reduces ESR1 mRNA and protein levels and inhibits its transcriptional activity^[135]^. In contrast, one report observed a pro-tamoxifen effect of HDAC4 in ER-positive MCF-7 and T47D cell lines. However, the mechanism by which HDAC4 directs such activity remains unclear^[136]^.

Androgen deprivation therapy (ADT) is the most important treatment for locally advanced and metastatic prostate cancer. Unfortunately, resistance very often occurs within a few years, leading to castration-resistant prostate cancer (CRPC)^[137]^. Multiple different mechanisms of alteration are engaged to strongly sustain the AR signaling and CRPC^[138]^.

The involvement of HDACs in ADT resistance has been described in several studies, and treatments with HDACi have been proposed to overcome drug resistance in CRPC^[139]^. One study demonstrated that the expression of androgen receptor (AR)-regulated genes is dependent on HDAC1 or HDAC3^[140]^, a finding later confirmed by another study^[141]^. Furthermore, it has been proposed that the activities of class I HDACs are necessary for the assembly of the coactivator/RNA polymerase II complex after AR binds to the enhancers of target genes^[140]^.

The orphan nuclear receptor TLX (NR2E1) is upregulated in prostate cancer, especially in metastatic CRPC. TLX can also induce resistance to androgen deprivation by suppressing AR transcription. HDAC1 and HDAC3 are important to propel this suppression^[142]^. To make the androgen response even more complex, HDAC3 can also suppress AR expression through the involvement of FOXO1^[143]^.

A functional screen identified eight candidate genes for AR-directed therapy resistance, which are mutated by APOBEC3B, including HDAC5^[144]^. Cytosine deaminase APOBEC is an important trigger of mutations in cancer. In prostate cancer, the synaptotagmin-binding cytoplasmic RNA-interacting protein (SYNCRIP), which suppresses APOBEC-dependent mutagenesis, is frequently lost. Its absence leads to resistance to AR-targeted therapies. Knockout (KO) of *HDAC5* has revealed that this class IIa HDAC is involved in maintaining resistance to the AR antagonist enzalutamide in prostate cells lacking SYNCRIP^[144]^. However, the precise mechanism by which HDAC5 contributes to this resistance, and the impact of the associated mutations, remains unclear. Considering the previous observation that HDAC4 can antagonize AR activity through SUMOylation, a similar contribution could be evoked for HDAC5. However, HDAC5 was much less efficient in supporting AR SUMOylation^[145]^. Nevertheless, these results are of interest and warrant further investigation.

## TARGET-DIRECTED THERAPY

Cancer cells are dependent on the dysregulation of certain signaling pathways for their vigorous growth and aggressiveness. In some cases, specific inhibitors have been developed, but resistance and relapse often occur after an initial positive response^[146-148]^. In the following, we discuss some examples of the involvement of HDACs in the resistance mechanism.

Among pathway inhibitors, MAPK kinase inhibitors and MEKi-based therapies have received FDA approval for some cancers^[149,150]^. The RAS/RAF/MEK/ERK signaling pathway [mitogen-activated protein kinase (MAPK)] is associated with tumor cell proliferation and survival, particularly in cancers that harbor BRAF and/or RAS mutations^[151]^.

Trametinib is a selective inhibitor of MEK1/2, which prevents RAF- and RAS-dependent MEK phosphorylation and thus inhibits ERK phosphorylation^[151]^. HDAC3 is thought to be involved in MEKI resistance in PDAC^[149]^. The NCor1/HDAC3 complex interacts with G9a, a Lys methyltransferase, and SETD5, a chromatin-associated protein with a catalytic methyltransferase SET domain, which instructs the complex to deacetylate H3K9ac, resulting in this Lys residue methylation at genes involved in MEKI resistance, such as *PDK4*^[152]^. The authors combined the treatment of HDAC3i and G9ai in trametinib-resistant cells, which restored MEKi sensitivity^[149]^.

Trametinib resistance has also been identified in NSCLC, and HDAC3 inhibition provides therapeutic benefits in a Kras-mutated/Lkb1-depleted mouse model of NSCLC^[153]^.

Other pathway inhibitors that have been studied in the context of HDACs-mediated resistance are tyrosine kinase inhibitors (TKIs), such as sunitinib, which acts as an anti-angiogenic agent and is used in particular for kidney cancer^[154]^. Resistance to these inhibitors could be due to proangiogenic signaling pathways, changes in the tumor microenvironment, increased tumor invasion and metastasis, microRNA-mediated drug resistance, or activation of other signaling pathways^[155]^.

ANXA1 is linked to sensitivity to TKIs^[156]^, and in clear cell renal cancer (CCRC), YTHDC1, a reader of the m6A modification^[157]^, interacts with ANXA1 mRNA via the m6A modification, reducing its stability, thereby inactivating the MAPK signaling pathway and rendering CCRC cells sensitive to TKIs^[158]^. HDAC2 in complex with TF YY1 suppresses the expression of YTHDC1, indirectly stabilizes ANXA1 mRNA, activates the MAPK pathway, and induces TKI resistance^[158]^.

Sorafenib is a multitarget TKI approved for the treatment of hepatocellular carcinoma (HCC). Sorafenib targets the RAF/MEK/ERK signaling pathway and receptor tyrosine kinases and inhibits tumor growth and progression^[159]^. A current problem of HCC patients is the development of sorafenib resistance, which occurs through different mechanisms, including epigenetics mechanisms, transport processes regulation, modulation of cell death, and alterations in the tumor microenvironment^[160,161]^. The TF MEF2D is one of the main partners of HDAC4^[162,163]^. It has been described that the influence of HDAC4 on sorafenib resistance depends on MEF2D. This TF is highly expressed in HCC patients with poor prognosis and is more abundant in sorafenib-resistant cells than in sensitive cells. HDAC4 forms a complex with MEF2D and directly regulates its activity on SPRY4, an inhibitor of the MAPK/ERK signaling pathway. The MAPK/ERK signaling pathway is a known player in sorafenib resistance^[164,165]^. Treatment with the HDAC4 inhibitor tasquinimod inhibits MEF2D-dependent suppression of SPRY4 and leads to re-sensitization to sorafenib in a liver cancer mouse model^[166]^. Of course, one must bear in mind that tasquinimod also has other targets and that specific activity against HDAC4 cannot be ruled out^[167]^.

## PALBOCICLIB

Palbociclib is a CDK4/6 inhibitor that is used in particular for the treatment of hormone-positive breast cancer. It inhibits the phosphorylation of the tumor suppressor gene RB and leads to cell cycle arrest. Resistance to palbociclib is an emerging problem caused both by a failure of the CDK4/6-RB axis and by changes in the upstream regulatory factors^[168,169]^. It is known that RB binds to HDAC1 via its pocket domain and an LXCXE motif in HDAC1^[170]^. Phosphorylation-dependent recruitment of HDAC1/2 is also utilized by RB to form a repressive complex^[171]^. Based on this historical evidence, one could argue that HDAC1/2 is necessary for the antiproliferative effect of palbociclib - a hypothesis that has not yet been investigated. Interestingly, enhancing the repressive activity of the RB1/TEAD4/HDAC1 axis leads to the suppression of DNA repair genes and sensitizes the cells to oxaliplatin treatment^[172]^. HDAC5 has also been reported to interact with the RB protein, and binding is reduced by CDK4/6-dependent phosphorylation and enhanced by palbociclib treatment. HDAC5 acts as a repressive arm of RB-dependent gene silencing by inhibiting H3K27ac at multiple oncogenic loci associated with the cell cycle^[173]^. In prostate and breast cancer cells, HDAC5 KD promotes resistance to palbociclib. Indeed, palbociclib is not sufficient to activate HDAC5-dependent RB inhibitory activity in HDAC5 KD cells, but concurrent treatment with NEO3724, a dual BET-CBP/p300 inhibitor, restores sensitivity to palbociclib in cancer cells and xenografts^[173]^. Of note, all class IIa HDACs, but not class I HDACs, appear to be involved in palbociclib-dependent inhibition of H3K27ac at cell cycle loci in prostate cancer cells. Zhou *et al.* also reported that the expression of HDAC5 is frequently reduced in various solid tumors, including breast and prostate cancer^[173]^. This observation is inconsistent with several reports indicating a positive correlation between class IIa HDACs and cancer aggressiveness. However, we need to consider the contextual influence that could explain the differential contribution of these epigenetic regulators to tumorigenesis in specific contexts. The author also used the inhibitor LMK235 to specifically demonstrate the contribution of HDAC5. However, this compound shows some preference but no specificity toward HDAC5^[174,175]^.

## PARP INHIBITORS

PARP inhibitors (PARPi) are effective in the treatment of ovarian and breast cancer, especially in cancers that are deficient in homologous recombination (HR) and DNA repair mechanisms due to BRCA1/2 mutations^[176]^. Nevertheless, cancer cells often develop resistance to PARPi, which is why combination therapies need to be developed^[177,178]^.

The influence of class I HDACs on DSB repair by HR suggests a possible contribution of these deacetylases to the efficacy of PARPi treatments^[18,179,180]^. In some cases, the contribution of specific class I inhibitors has been explored with positive results in preclinical models of ovarian cancer and one study suggested a new role for HDAC2 in the regulation of splicing of the HR genes *BRCA1/FANC*^[181,182]^.

Class IIa HDACs can also affect DNA repair pathways and HR. Although the detailed mechanisms remain to be fully explored, an effect through the binding of members of the MEF2 family or through the regulation of chromatin accessibility in complex with class I HDACs has been proposed^[18,183-185]^.

Class IIa HDACs interact with MEF2 at the level of the N-terminal domain within the nucleus when HDACs IIa members are not phosphorylated at 14-3-3 binding sites^[8,22,186]^. Salt-inducible kinase 2 (SIK2), an AMPK-related protein kinase that has a positive effect on ovarian cancer progression, phosphorylates HDACs IIa, causing its cytoplasmic localization and inhibiting its binding to MEF2^[187,188]^. The SIK2 inhibitors ARN3236 and ARN3261 prevent HDAC4/HDAC5 phosphorylation, resulting in their nuclear localization and inhibition of the MEF2D transcriptional program. ARN3236 and ARN3261 were found to repress transcription of DNA repair genes such as *EXO1*, *FANCD1*, and *XRCC4* due to inhibition of MEF2D by class IIa HDACs and restore PARPi sensitivity in ovarian cancer and TNBC cells^[178]^.

## REVERSAL OF RESISTANCE: NEW PERSPECTIVES FOR HDAC INHIBITORS

A logical perspective in defining the contribution of HDACs to drug resistance is their targeting by specific inhibitors. The first frontier stems from biology. Our current knowledge of the contribution of HDACs to resistance is not clear and adverse effects cannot be excluded. We must assume that under certain circumstances, these enzymes play a role in determining the effect of drug treatment. The second limit concerns inhibitors. We have had experience with HDACi for more than two decades, but the clinical applications are few and limited to a pair of hematologic cancers^[9]^. The first generation of non-selective HDACi has failed in several clinical trials alone or in combination. The major problems are lack of selectivity, various off-targets, and toxicity. These non-selective HDACi include clinically approved drugs such as vorinostat/SAHA, romidepsin/FK228, belinostat PXD101, and panobinostat/LBH589. They act as zinc chelators by penetrating the catalytic pocket of HDACs^[189]^. The efficacy of these HDACi in the treatment of solid tumors as single agents was disappointing^[190,191]^. Some results have been reported when used in combination therapy and particularly with immunotherapy, with some clinical trials still ongoing^[192]^.

Subsequently discovered family- or member-specific inhibitors may offer more effective options as they might reduce toxicity and side effects observed with the first generation of non-selective HDACi. These include entinostat/MS-275 and NKL54, which favor class I enzymes other than HDAC8, and chidamide/CS055, which has been approved by the China Food and Drug Administration for peripheral T-cell lymphoma and advanced breast cancer^[189]^. The future perspective for HDACi lies in the identification of new isoform-selective and more effective inhibitors. These new agents could intervene more specifically to overcome cancer-related drug resistance, where the specific HDAC isoform determines the resistance phenotype. Innovative approaches such as proteolysis targeting chimera (PROTAC) also need to be utilized, although delivery of PROTAC-derived molecules to cancer tissue (due to large molecular mass and polarity) is an important limitation^[193]^. Less explored approaches such as miRNA-based therapies that mimic the various natural mRNAs that target these HDACs^[22,194-198]^ or the development of small compounds that act as allosteric drugs to modify the assembly of the multiprotein complexes containing HDACs deserve special investigation.

Gene silencing occurs through the coordinated activities of various epigenetic regulators acting on different histone PTMs and DNA methylation. Therefore, concomitant treatment with inhibitors targeting other epigenetic regulators that may act synergistically with HDACs to suppress gene expression should be considered, especially if the resistant phenotype is due to epigenetic dysregulation. For example, DNMT inhibitors may synergize strongly with HDACi and activate gene expression differently than single-agent treatments^[199-202]^. Of particular interest is the recent development of dual DNMT1/HDAC inhibitors, which may have a similar synergistic effect to combined treatment with the two inhibitors, but with less toxicity^[203]^.

## CONCLUSION

The contribution of HDACs to drug resistance, particularly the zinc-dependent HDACs forming multiprotein complexes discussed in this review, has been investigated in different tumors and under different treatments. Over the last decade, evidence has accumulated for the contribution of HDACs to drug resistance in cancer. In general, their role in promoting resistance is predominant [Tables 1 and 2]. This has opened up the possibility of reversing the resistant phenotype with HDACi. In some studies, non-selective HDACi such as vorinostat/SAHA have been used and their effect could not be focused on the specific alteration. More recently, family-specific inhibitors have entered the market, potentially offering a more efficient means of intervention.

**Table 1:** Summary of the contribution of class I HDACs (HDAC1/2/3) to drug resistance or (required) for drug action in the specified cancer type

**HDAC**	**Drug**	**Cancer type**	**Contribution**	**Ref.**
HDAC1	Cisplatin	Breast cancer	Required	[36]
	Cisplatin	Laryngeal cancer	Resistance	[39]
	Cisplatin	NSCLC	Resistance	[40]
	BTZ	MM	Resistance	[58]
	5-FU	CRC	Required	[79]
	PTX	NSCLC	Resistance	[91]
	DTX	LAD	Resistance	[99,100]
	ADT	Prostate cancer	Resistance	[142]
	Oxaliplatin	Rectal cancer	Required	[172]
	Olaparib	Ovarian cancer	Required	[181]
HDAC2	Cisplatin	Ovarian cancer	Required	[43]
	5-FU	CRC	Required	[79]
	Tamoxifen	Breast cancer	Resistance	[129]
	Sunitinib	CCRC	Resistance	[158]
	Olaparib	Ovarian cancer	Required	[181]
HDAC3	Cisplatin	Lung cancer	Required	[44]
	Doxorubicin	AML	Resistance	[65]
	DTX	Maxillary cancer	Resistance	[101]
	DTX	Prostate cancer	Required	[102]
	Exemestane	Breast cancer	Resistance	[126]
	ADT	Prostate cancer	Resistance	[142]
	Trametinib	PDAC	Resistance	[149]
	Trametinib	NSCLC	Resistance	[153]

HDACs: Histone deacetylases; NSCLC: non-small cell lung cancer; BTZ: bortezomib; MM: multiple myeloma; 5-FU: 5-fluorouracil; CRC: colorectal cancer; PTX: paclitaxel; DTX: docetaxel; LAD: lung adenocarcinoma; ADT: androgen deprivation therapy; CCRC: clear cell renal cancer; AML: acute myeloid leukemia; PDAC: pancreatic ductal adenocarcinoma.

**Table 2:** Summary of the contribution of class IIa HDACs to drug resistance or drug action (required) in the specified cancer(s)

**HDAC**	**Drug**	**Cancer type**	**Contribution**	**Ref.**
HDAC4	Cisplatin	Ovarian cancer	Resistance	[45]
	Cisplatin	Gastric cancer	Resistance	[46]
	Doxorubicin	Lung cancer	Resistance	[69]
	Methotrexate	Osteosarcoma and CRC	Required	[82]
	5-FU	Osteosarcoma	Required	[82]
	5-FU	CRC	Resistance	[83]
	Gemcitabine	PDAC	Resistance	[84]
	PTX	PDAC	Resistance	[84]
	DTX	Lung cancer	Resistance	[100]
	Tamoxifen	Breast cancer	Required	[136]
	Sorafenib	HCC	Resistance	[166]
	PARPi	Breast and ovarian cancer	Required	[178]
HDAC5	Cisplatin	Ovarian cancer	Resistance	[53]
	Tamoxifen	Breast cancer	Resistance	[129]
	ADT	Prostate cancer	Resistance	[144]
	Palbociclib	Prostate cancer	Required	[173]
	PARPi	Breast and ovarian cancer	Required	[178]
HDAC7	5-FU	Lung cancer	Resistance	[81]
	Gemcitabine	PDAC	Resistance	[84]
	PTX	PDAC	Resistance	[84]
HDAC9	Tamoxifen	Breast cancer	Resistance	[135]
MITR	PTX	Breast cancer	Resistance	[104]

HDACs: Histone deacetylases; CRC: colorectal cancer; 5-FU: 5-fluorouracil; PDAC: pancreatic ductal adenocarcinoma; PTX: paclitaxel; DTX: docetaxel; HCC: hepatocellular carcinoma; PARPi: poly ADP-ribose polymerase inhibitors; ADT: androgen deprivation therapy; MITR: MEF-2 interacting transcription repressor.

Frequently, alterations in HDACs associated with drug resistance occur (and have been studied) in the context of highly mutated cancer cell lines. These cells have also accumulated alterations in other key genes associated with drug resistance, including elements of the apoptotic machinery, the DDR, or elements controlling drug metabolism and availability. For these reasons, a combination therapy targeting some of these altered cellular responses should be used.

**Figure 3:**
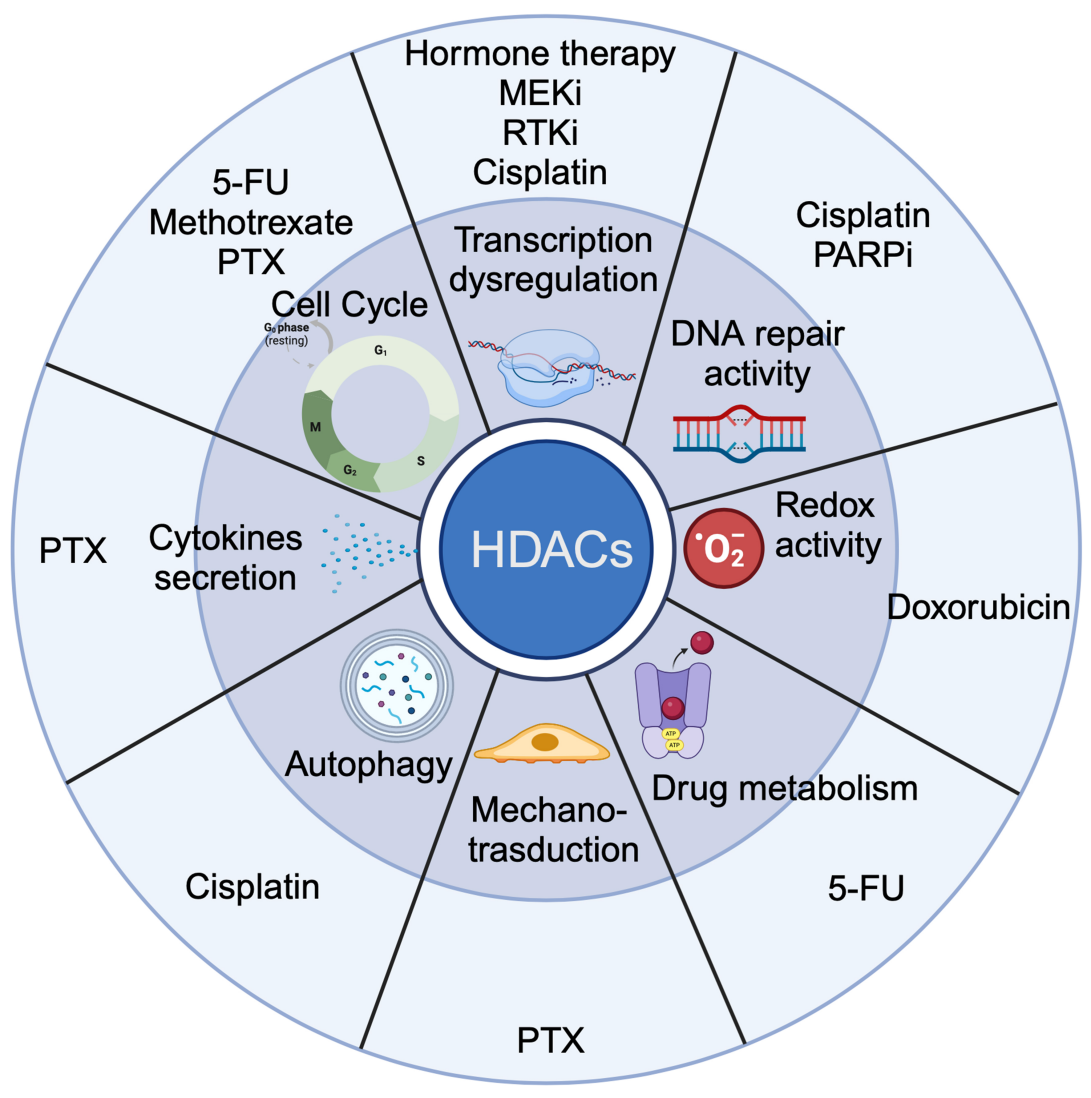
Summary of the different cellular processes regulated by HDACs (1/2/3/4/5/7/9) that may determine anticancer drug resistance. Dysregulation of transcription can also affect the other biological processes mentioned. The different treatment options are outlined. HDACs: Histone deacetylases.

There are many examples of the contribution of these HDACs to the failure of specific cancer therapies with genetically validated results, which are summarized in Figure 3. However, we have very little information about the specific complexes involved in the specific context. This is particularly critical for HDAC1/2, where different multiprotein complexes have been mapped and defined. The contribution of the different complexes characterized by the deacetylase activities of class I HDACs could explain the large heterogeneity of mechanisms described in the literature under the supervision of these HDACs (class I and IIa). We have more differences than confirmations. Although the contribution of HDACs to resistance predominates, there are also cases where they are essential for treatment efficacy [Tables 1 and 2]. These contrasting results should not be a surprise. The context of specific cancer-associated changes and (again) the assembly of HDACs in different multiprotein complexes, whose composition may depend on cancer, are logical hypotheses to explain such differences.

Although HDACs are important epigenetic regulators and important examples of changes in histone acetylation status associated with therapy resistance have been documented, there are other cases involving the regulation of non-histone proteins^[45,49,50,53,65,81,84,130]^. Certainly, their contribution as epigenetic regulators is underestimated. In particular, their role in shaping H3K27ac at enhancers and super-enhancers and how this contributes to drug resistance is poorly studied^[204-211]^. An obvious aspect that we should also consider is the relationship with the antagonizing enzymes, the KATs, both in terms of contribution to drug resistance and possible targets to improve therapy depending on the specific environment.

The ultimate goal of these studies should be to gain insight into the contribution of HDAC-dependent changes in a patient-specific manner and thus stratify patients to optimize therapeutic strategy. This would optimize benefits for patients and costs for healthcare systems. Of course, the question is not easy to answer, as the nature (point mutations, changes in expression, different PTMs, assembly into different multiprotein complexes, *etc.*) and the number of alterations can vary greatly. However, since these HDACs are primarily epigenetic regulators, a defined signature of changes in gene expression could be a possible indirect method for unmasking their alterations.

## DECLARATIONS

### Copyright

© The Author(s) 2024.
